# Ischemic Injury-Induced CaMKIIδ and CaMKIIγ Confer Neuroprotection Through the NF-κB Signaling Pathway

**DOI:** 10.1007/s12035-018-1198-2

**Published:** 2018-07-11

**Authors:** Jing Ye, Sabyasachi Das, Adhiraj Roy, Wenzhong Wei, Huachen Huang, Joshua Michael Lorenz-Guertin, Qian Xu, Tija C. Jacob, Bing Wang, Dandan Sun, Qiming Jane Wang

**Affiliations:** 1 Department of Pharmacology and Chemical Biology, University of Pittsburgh School of Medicine, E1354 BST, Pittsburgh, PA 15261 USA; 2 Department of Anesthesiology, Nanfang Hospital, Southern Medical University, Guangzhou, People’s Republic of China; 3 Department of Orthopaedic Surgery, University of Pittsburgh, Pittsburgh, PA USA; 4 Department of Neurology, University of Pittsburgh, Pittsburgh, PA USA; 5 Department of Neurology, The First affiliate Hospital, Harbin Medical University, Harbin, Heilongjiang China; 6 China-UK-NYNU-RRes Joint Laboratory, Nanyang Normal University, Nanyang, People’s Republic of China

**Keywords:** Stroke, In vitro ischemia, CaMKII, Long non-coding RNA, Neuroprotection

## Abstract

Ca^2+^/calmodulin-dependent protein kinase II (CaMKII) has long been implicated in neuronal injury caused by acute ischemia/reperfusion (I/R). However, its precise role and regulatory mechanisms remain obscure. Here, we investigated the role of the CaMKII family in neuronal survival during I/R. Our data indicated that *CAMK2D*/CaMKIIδ and *CAMK2G*/CaMKIIγ were selectively upregulated in a time-dependent manner at both transcriptional and protein levels after acute ischemia. Overexpression of CaMKIIδ promoted neuronal survival, while their depletion exacerbated ischemic neuronal death. Similar to CaMKIIδ, knockdown of CAMKIIγ resulted in significant neuronal death after I/R. We further identified CaMKIIδ_2_ as the subtype that is selectively induced by I/R in primary neurons. The induction of CaMKIIδ was controlled in part by a pair of long non-coding RNAs (lncRNAs), *C2dat1* and *C2dat2*. *C2dat2*, similar to *C2dat1*, was upregulated by I/R and cooperated with *C2dat1* to modulate CaMKIIδ expression. Knockdown of *C2dat1/2* blocked OGD/R-induced CaMKIIδ expression and decreased neuronal survival but did not affect the levels of CaMKIIγ, indicating specific targeting of *CAMK2D* by *C2dat1/2*. Mechanistically, I/R-induced CaMKIIδ and CaMKIIγ caused the upregulation of IKKα/β and further activation of the NF-κB signaling pathway to protect neurons from ischemic damage. Genetically, downregulating p65 subunit of NF-κB in mice increased I/R-induced neuronal death by blocking the activity of CaMKII/IKK/IκBα/NF-κB signaling axis. In summary, CaMKIIδ and CaMKIIγ are novel I/R-induced genes that promote neuronal survival during ischemic injury. The upregulation of these CaMKII kinases led to activation of the NF-κB signaling pathway, which protects neurons from ischemic damage.

**Electronic supplementary material:**

The online version of this article (10.1007/s12035-018-1198-2) contains supplementary material, which is available to authorized users.

## Introduction

Stroke is a worldwide health problem that leads to high rates of death and neurological disability in adults. Focal cerebral ischemia is a type of stroke that inflicts brain damages by reducing blood flow and halting oxygen supply to the brain. The mechanisms underlying cerebral ischemia injury are complex and still less understood. Despite the therapeutic advances in recent years, there remain limited treatment options for this disease.

The family of calcium/calmodulin-dependent kinase II (CaMKII) comprises of four isoforms (CaMKIIα, β, γ, δ) encoded by different genes that display distinct and overlapping expression [1–3]. The CaMKII holoenzyme is a homo- or hetero-multimer that is assembled by 8 to 12 isoforms. It is by far the most abundant protein kinase in the brain. CaMKII is multifunctional and plays important roles in synaptic transmission and plasticity that affect behavior, learning, and memory. At molecular level, it modulates a wide range of cellular processes including calcium (Ca^2+^) homeostasis, gene transcription, receptor function, and cytoskeletal alterations [1, 4–9]. It has been implicated in both neuronal death and survival, and its precise role in brain ischemia remains to be determined. I/R injury induces many genes and pathways involved in inflammation, apoptosis, and oxidative stress [10]. Among them, the nuclear factor-κB (NF-κB) signaling pathway is a major mediator of these complex biological processes, and dysregulation of NF-κB transcriptional activity has been linked to neurodegenerative diseases and numerous inflammatory conditions [11]. The regulatory mechanisms and functions of the NF-κB pathway remain to be fully elucidated in cerebral ischemia.

The regulation of individual CaMKII isoform expression and activity is complex and not fully understood. We have previously reported a novel *CAMK2D*-associated long non-coding RNA (lncRNA) – *C2dat1* in neurons*. C2dat1* is induced by I/R and targets *CAMK2D* for upregulation in murine neuronal cells and mouse I/R models. Increased CaMKIIδ promotes neuronal survival by activating the NF-κB signaling pathway [12]. Compared to CaMKIIδ, *CAMK2G*/CaMKIIγ is a less-known member of the CaMKII family and its role in the CNS has just begun to emerge. It has been shown that the upregulation of CaMKIIγ facilitates local anesthetic-induced nerve injury [13]. CaMKIIγ transports sequestered Ca^2+^/calmodulin from the neuronal surface to the nucleus and triggers a highly cooperative activation of the nuclear CaMK cascade [5, 14]. Taken together, despite intense studies on CaMKII holoenzyme in post-insult neurons, its precise role, signaling mechanisms, and the specific isoforms involved in the process remain to be fully defined.

In this study, we conducted a thorough analysis on the CaMKII family kinases in murine neuronal cells and primary neurons subjected to I/R or in vitro ischemia. Our study revealed selective induction of CaMKIIδ and CaMKIIγ genes and proteins by ischemia. The upregulation of CaMKIIγ and CaMKIIδ acts to protect neurons from ischemic damage. We found that two novel lncRNAs, *C2dat1* and *C2dat2*, mediated ischemia-induced upregulation of CaMKIIδ. Further, CaMKIIδ and CaMKIIγ activated the NF-κB signaling pathway to promote neuronal survival. This study shed lights to neuroprotective mechanisms evoked after ischemic injury in the brain.

## Results

### Ischemia/Reperfusion (I/R) Induced Time-Dependent Upregulation of *CAMK2D*/CaMKIIδ and *CAMK2G*/CaMKIIγ

**Fig. 1:**
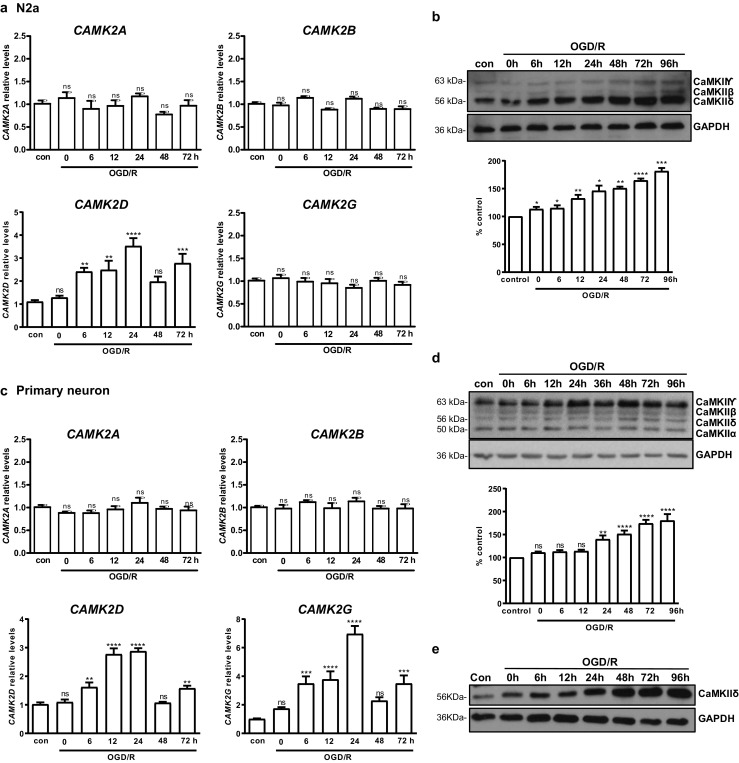
OGD/R induced *CAMK2D*/CaMKIIδ and*CAMK2G*/CaMKIIγ upregulation in vitro and in vivo. N2a cells (**a**, **b**) or rat primary neurons (**c**, **d**) were subjected to OGD for 3 h (N2a) or 1 h (primary neurons), followed by reoxygenation for indicated times. The cells were collected and analyzed by real-time RT-qPCR for transcripts of *CAMK2A*, *CAMK2B*, *CAMK2D*, and *CAMK2G* (**a**, **c**) and by Western blotting using anti-CaMKII (pan) (D11A10) antibody (**b**, **d**, top). Levels of CaMKIIδ were quantified by densitometry analysis (**b**, **d**, bottom). Cells cultured in normal medium under normoxic condition were used as controls (con). Percent control (*y*-axis) represents the expression of CaMKIIδ (**b**) or CaMKIIγ (**d**) to that of the controls (equal to 100%). **e** Rat primary neurons were subjected to OGD/R, followed by immunoblotting using a CaMKIIδ-specific antibody. The data in **a** and **c** are the mean ± SEM of five independent experiments with triplicate determinations at each point. Representative images from one of four independent experiments are shown in **b**, **d**, and **e**. Densitometry data are the mean ± SEM of four independent experiments. ns, not significant; **P < 0.01; ***P < 0.001; ****P < 0.0001 vs. control by one-way ANOVA. con control

We evaluated the effects of I/R on the expression of CaMKII kinases. Mouse Neuro 2A (N2a) cells and rat/mouse primary neurons were subjected to in vitro ischemia by oxygen-glucose deprivation followed by reoxygenation (OGD/R). The cells were collected at different time points of reoxygenation, and the levels of *CAMK2A*, *CAMK2B*, *CAMK2D*, and *CAMK2G* mRNAs were measured by real-time RT-qPCR and Western blotting. In N2a cells, at the basal state, *CAMK2G* and *CAMK2D* mRNAs were expressed at higher levels as compared to *CAMK2A* and *CAMK2B* (Fig. S1A). In response to OGD/R, an over 3-fold upregulation of *CAMK2D* was observed, which peaked at 24 h and returned to baseline at 48 h, followed by a 2nd peak at 72 h. In contrast, we did not observe any changes of *CAMK2A*, *CAMK2B*, and *CAMK2G*. At the protein level, CaMKIIδ protein was detected as the predominant CaMKII isoform in N2a cells using a pan-CaMKII antibody, while the expression of CaMKIIγ and CaMKIIβ were lower and there was little/no CaMKIIα in N2a cells (Fig. 1b). Upon OGD/R treatment, we observed a persistent and time-dependent upregulation of CaMKIIδ (1.5~2-fold) using the pan-CaMKII antibody (Fig. 1b). The expression of CaMKIIδ was further verified using an isoform-specific CaMKIIδ antibody, which demonstrated significant expression of CaMKIIδ in N2a cells (Fig. S1B), re-affirming the findings obtained using the pan-CaMKII antibody.

In contrast to N2a, in rat primary cortical neurons, *CAMK2G* and *CAMK2A* mRNAs were detected at higher levels as compared to *CAMK2B* and *CAMK2D* at the basal state (Fig. S1D). In response to OGD/R, a time-dependent upregulation of *CAMK2D* (~ 3-fold) was similarly detected, which peaked at 12 and 24 h and returned to baseline at 48 h, followed by another peak at 72 h. Additionally, distinct from N2a cells, we also observed an approximately 7-fold increase of *CAMK2G* that peaked at 24 h. No changes of *CAMK2A* and*CAMK2B* were observed (Fig. 1c). At the protein level, CaMKIIγ was the predominant CaMKII isoform detected using the pan-CaMKII antibody, while the levels of other isoforms were lower in primary neurons (Figs. 1d and S1D). In accordance with increased *CAMK2G*, CaMKIIγ was persistently upregulated over time. There was also a significant increase of CaMKIIδ detected by the pan-CaMKII antibody, which was validated using a CaMKIIδ-selective antibody in primary neurons (Fig. 1e). In contrast, no changes of CaMKIIα and CaMKIIβ were observed in neurons (Fig. 1d). Collectively, our data indicated that OGD/R selectively induced the upregulation of *CAMK2D/*CaMKIIδ and *CAMK2G*/CaMKIIγ in primary neurons, implying a potential role of these kinases in ischemic-associated biological processes in neuronal cells.

**Fig. 2:**
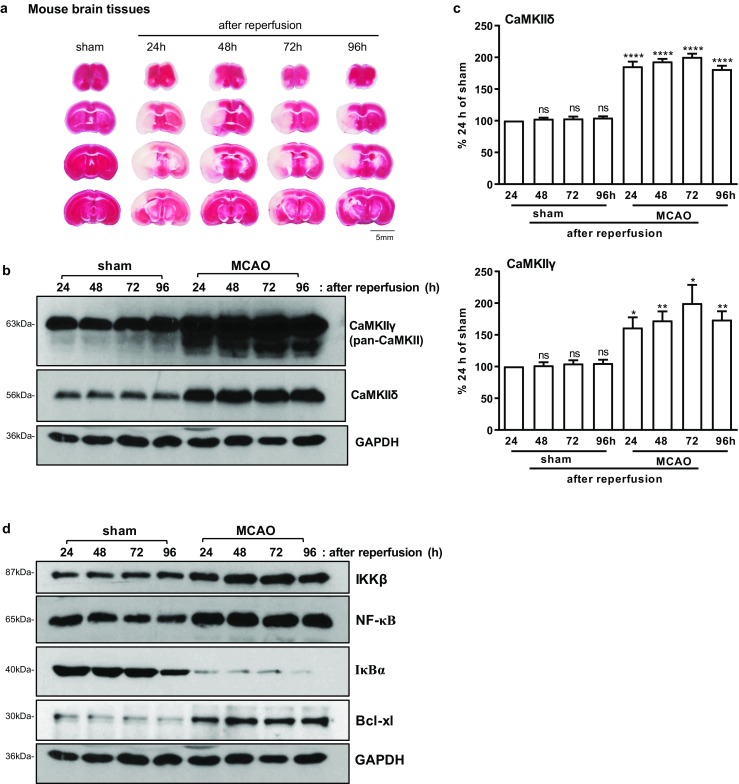
I/R induced CAMK2D/CaMKIIδ andCAMK2G/CaMKIIγ upregulation in mice. **a** Representative coronal brain sections stained with 2,3,5-triphenyltetrazolium chloride monohydrate (TTC) are shown. Cerebral infarction induced by MCAO was visible at 24, 48, 72, and 96 h after reperfusion. **b** Analysis of CaMKIIγ and CaMKIIδ expression in penumbra of sham and ischemic (MCAO) tissues by Western blotting. **c** Quantitative analysis of CaMKIIγ and CaMKIIδ by densitometry. % Control was calculated with sham (24 h) as 100%. Data are the mean ± SEM from four independent experiments. ns, not significant; *P < 0.05; **P < 0.01; ****P < 0.0001 vs. control by one-way ANOVA. con, control. **d** Western blotting analysis of the NF-κB signaling pathway in penumbra of sham and MCAO tissues. Representative images from one of four independent experiments are shown in **b** and **d**

Next, focal cerebral ischemia was induced in mice by intraluminal occlusion of the left middle cerebral artery (MCAO) for 1 h, followed by prolonged reperfusion (24, 48, 72, and 96 h) where neural protection and recovery are thought to take place. As shown in Fig. 2a, MCAO induced irreversible infarction visible 24 h with progressive neuronal death in the ischemic core peaked at 72 h, followed by a recovery period visible at 96 h. We detected sustained upregulation of CaMKIIδ and CaMKIIγ at 24, 48, 72, and 96 h in brain tissues collected from the surrounding penumbra (Fig. 2b, c), which was accompanied by increased IKKβ and NF-κB levels, degradation of IκBα, and induction of the pro-survival NF-κB target gene Bcl-xL, indicating potent activation of the NF-κB signaling pathway (Fig. 2d). Thus, I/R-induced upregulation of CaMKIIδ and CaMKIIγ was a physiological relevant event that coupled with the NF-κB signaling pathway in vivo.

### Differential Expression and Induction of *CAMK2D* Subtypes by OGD/R in N2a Cells and Primary Neurons

**Fig. 3:**
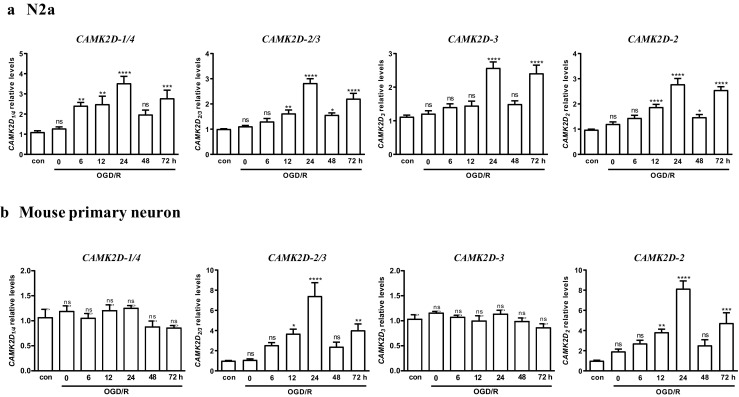
*CAMK2D* subtypes induced by OGD/R in N2a cells and primary neurons. N2a cells (**a**) or mouse primary neurons (**b**) were subjected to OGD for 3 h (N2a) or 1 h (primary neurons), followed by reoxygenation for indicated times. The cells were collected and analyzed by real-time RT-qPCR for *CAMK2D*_1/4_, *CAMK2D*_3_, *CAMK2D*_2/3_,and *CAMK2D*_2_ transcripts. The mean ± SEM of five independent experiments with triplicate determinations were calculated at each point. ns, not significant; *P < 0.05; **P < 0.01; ***P < 0.001; ****P < 0.0001 vs. control by one-way ANOVA. con, control

Alternative splicing of *CAMK2D* gives rises to at least ten subtypes of *CAMK2D* genes. In order to determine the specific *CAMK2D* isotypes induced by I/R, we designed real-time PCR primers that can selectively detect subtypes -1/4, -2/3, -2, and -3 of *CAMK2D*, namely*CAMK2D*_1/4_, *CAMK2D*_2/3_, *CAMK2D*_2_, and *CAMK2D*_3_. Using these primers, we then examined the mRNA levels of *CAMK2D* subtypes in response to OGD/R. Our data indicated that all four subtypes of *CAMK2D* were detected in N2a cells, and all were induced by OGD/R in similar kinetics, which peaked at about 24 h, declined in 48 h, followed by a 2nd peak at 72 h re-oxygenation (Fig. 3a). In contrast, in mouse primary neurons, the upregulation was only detected for *CAMK2D*_2/3_ and *CAMK2D*_2_, but not for *CAMK2D*_1/4_ and*CAMK2D*_3_, indicating that *CAMK2D*_2_ was the only subtype that was selectively upregulated by I/R (Fig. 3b).

### CaMKIIδ and CaMKIIγ Promoted Neuronal Survival in Response to In Vitro Ischemia

**Fig. 4:**
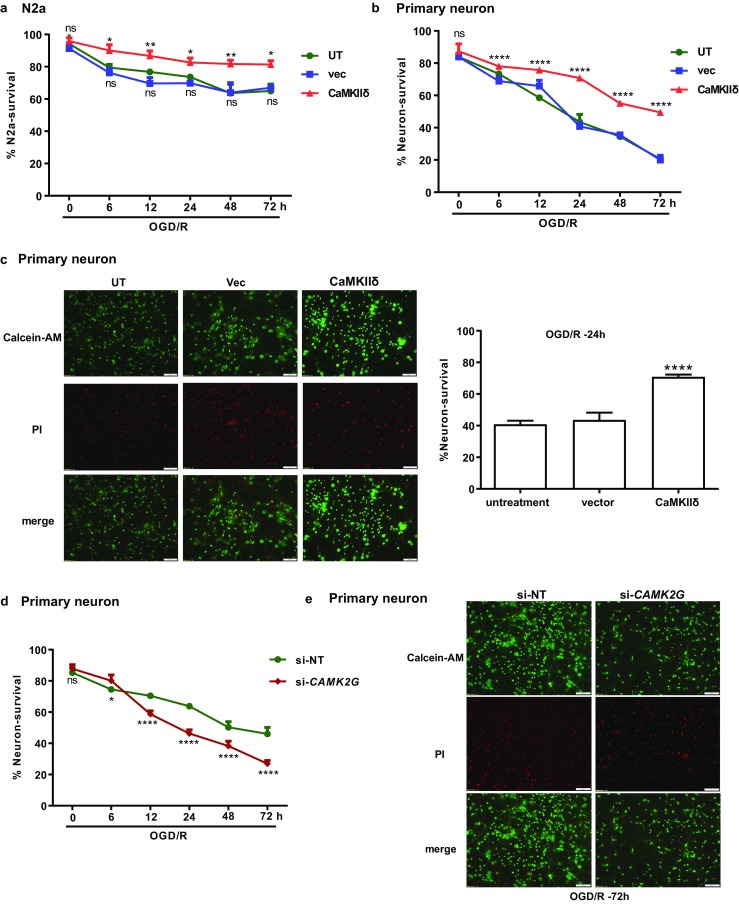
CaMKIIδ and CaMKIIγ protected neurons from OGD/R-induced cell death. **a**, **b** CaMKIIδ overexpression promoted cell survival in N2a cells (**a**) and primary neuronal cells (**b**). N2a and primary neuronal cells were transfected with the Myc-DDK-CaMKIIδ plasmid or empty vector. After 24 h, the cells underwent OGD/R treatment as described. The untransfected cells (UT) and cells transfected with the empty control vector (vec) are used as controls. Cell survival was determined by CCK-8 assay (N2a) or assessed by Calcein-AM/PI staining (primary neuron). The data in **a** are mean ± SEM from five independent experiments. **c** Images of calcein-AM/PI-stained primary neurons at OGD/R-24 h. red, PI; green, calcein-AM. Quantitative measures of live neurons (green) are shown (right). **d** Knockdown of *CAMK2G* increased OGD/R-induced cell death in primary neuronal cells. Primary neuronal cells were transfected with *CAMK2G* siRNA (si-*CAMK2G*) or non-targeting siRNA (si-NT). After 48 h, the neurons were subjected to OGD/R. Cell survival was assessed by calcein-AM/PI staining. **e** Images of calcein-AM/PI-stained primary neurons at OGD/R-72 h. red, PI; green, calcein-AM. The images were obtained by an Olympus 1X71 inverted system microscope and analyzed by ImageJ software. The total PI-positive or calcein-AM-positive cells were counted from ten random fields in each image. The % neuron survival was calculated as the ratio of calcein-AM-positive cell number over total cell number. Quantitative data in **b**–**d** are the mean ± SEM from at least three independent experiments. ns, not significant; *P < 0.05; **P < 0.01; ****P < 0.0001 vs. UT by two-way ANOVA

To determine the functional significance of I/R-induced CaMKIIδ, N2a and primary neuronal cells were transfected with or without the Myc-DDK-CaMKIIδ plasmid (CaMKIIδ), followed by assessment of cell viability by calcein-AM/(propidium iodide) PI staining. For the control cells that were either untransfected (UT) or transfected with an empty vector (vec), OGD/R treatment resulted in progressive neuronal cell death that maximized at about 48 h (30% cell death), in line with previous reports [12]. In contrast, overexpression of CaMKIIδ significantly enhanced cell survival in both N2a (Fig. 4a) and primary neurons (Fig. 4b, c). At 24 h, nearly 80% neurons were viable as compared to only 40% in control neurons (UT and Vec) (Fig. 4c). These data indicated the overexpression of CaMKIIδ protected neurons from ischemia-induced cell death.

The effect of CaMKIIγ on neuronal survival was determined by knockdown of endogenous *CAMK2G* using *CAMK2G* siRNA (si-*CAMK2G*) in rat primary neurons. As shown in Fig. 4d, e, knockdown of *CAMK2G* enhanced ischemic cell death after OGD/R as compared to the control cells transfected with non-targeting siRNA (si-NT). Thus, similar to CaMKIIδ, the induction of CaMKIIγ by I/R also protected neurons from ischemic damage.

### Ischemia-Induced *C2dat1* and *C2dat2* lncRNAs Were Required for Upregulation of *CAMK2D*, but not *CAMK2G*, in Neuronal Cells

**Fig. 5:**
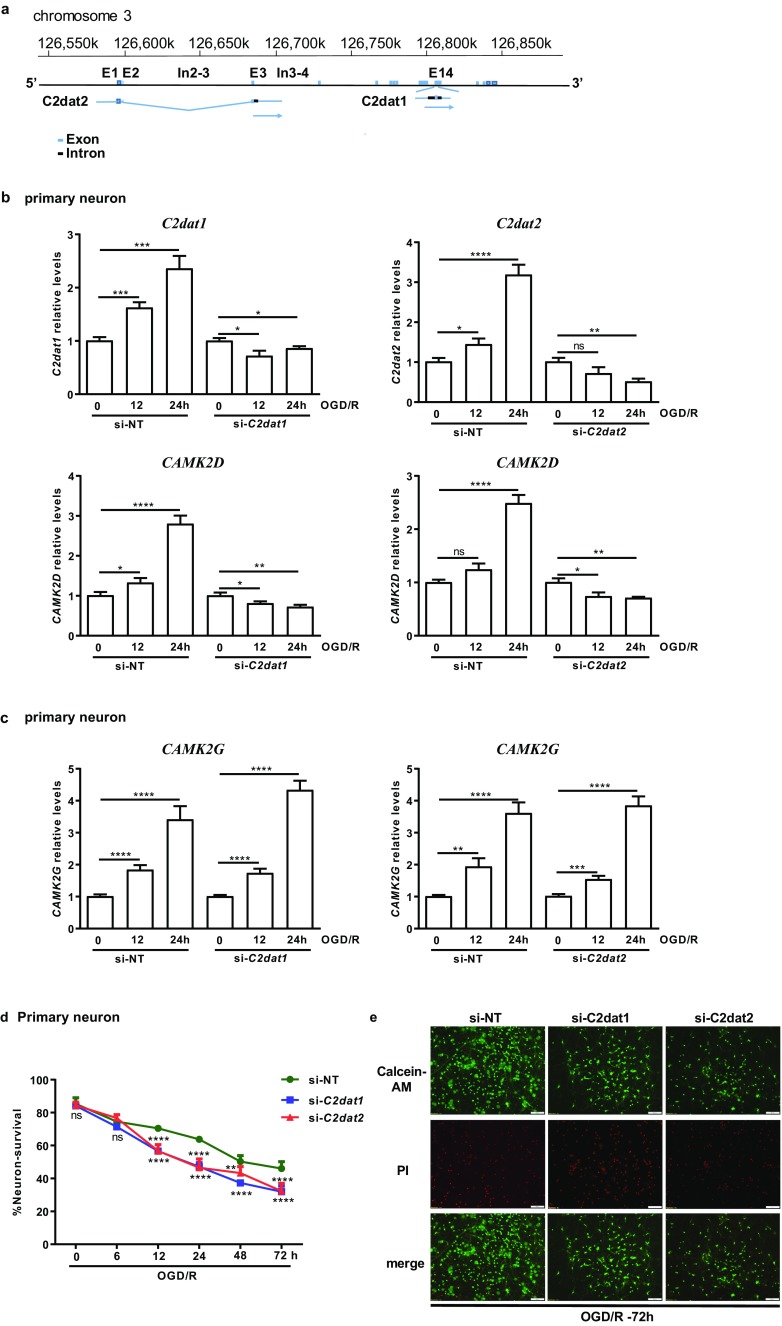
Knockdown of *C2dat1* or *C2dat2* blocked OGD/R-induced *CAMK2D* upregulation and exacerbated OGD/R-induced neuronal cell death. **a** Genomic locus of *C2dat1* and *C2dat2*. *C2dat2* contains overlapping sequences with introns 3–4 and exons 1–3 of *CAMK2D* on mouse chromosome 3, while *C2dat1* contains sequences from introns 13–15 and exon 14 of *CAMK2D*. **b**, **c** Knockdown of *C2dat1* or *C2dat2* inhibited OGD/R-induced upregulation of *C2dat1* or *C2dat2* and abolished OGD/R-induced *CAMK2D* expression (**b**), but did not block OGD/R-induced *CAMK2G* upregulation (**c**) in primary neuronals. lncRNA *C2dat1*- or *C2dat2*-targeted siRNA (si-*C2dat1*, si-*C2dat2*) and the non-targeting siRNA (si-NT) were transfected into primary neurons. Forty-eight hours after transfection, the cells were subjected to OGD/R for 0, 12, and 24 h. The cells were collected and analyzed by real-time RT-qPCR for *C2dat1*, *C2dat2*, *CAMK2D*, and *CAMKG* transcripts. The data are the mean ± SEM of at least three independent experiments with triplicate determinations at each point. ns, not significant; *P < 0.05; **P < 0.01; ***P < 0.001; ****P < 0.0001 vs. 0 h control by unpaired *t* test. **d** Primary neurons were transfected with si-*C2dat1*, si-*C2dat2*, or si-NT. Forty-eight hours later, the cells were treated with OGD/R. Cell survival was assessed by calcein-AM/PI staining. The total PI-positive or calcein-AM-positive cells were counted from ten random fields in each image. The % neuron survival was calculated as the ratio of calcein-AM-positive cell number over total cell number. The data are the mean ± SEM from at least three independent experiments. ns, not significant; **P < 0.01; ****P < 0.0001 vs. si-NT by two-way ANOVA. **e** Representative images of calcein-AM/PI-stained primary neurons at 72 h post-OGD/R. red, PI; green, calcein-AM

Our previous study described the identification of an intragenic lncRNA – *C2dat1* that overlapped in sequence with introns 13–15 and exon 14 of *CAMK2D* on mouse chromosome 3. *C2dat1* has been shown to modulate CaMKIIδ expression in response to I/R [12]. Here, we report the identification of a 2nd novel *CAMK2D*-associated lncRNA, *CAMK2D*-associated transcript 2 (*C2dat2*). The known sequence of lncRNA *C2dat2* overlaps in part with introns 3–4 and exons 1–3 of *CAMK2D* on mouse chromosome 3 (Fig. 5a). The overlapping sequences suggest that *C2dat2* may also regulate *CAMK2D*/CaMKIIδ expression. To test this hypothesis, *C2dat2* siRNA was designed to target the region of the lncRNA outside of the overlapped exon sequences with *CAMK2D*. N2a cells and rat primary neuronal cells were transfected with a *C2dat2*-targeting siRNA (si-*C2dat2*) which resulted in a significant knockdown of OGD/R-induced *C2dat2* in primary neurons (Fig. 5b) and N2a cells (Fig. S2A)*.* Importantly, knockdown of *C2dat1* or *C2dat2* significantly blocked OGD/R-induced *CAMK2D* expression in primary neurons (Fig. 5b) and in N2a cells (Fig. S2A). In contrast, levels of OGD/R-induced *CAMK2G* were not affected by the knockdown of *C2dat1* or *C2dat2* (Fig. 5c), demonstrating specific targeting of *CAMK2D*, but not other *CAMK2* isoforms, by *C2dat1* and *C2dat2*. These data also showed that knockdown of either lncRNAs completely abolished I/R-induced *CAMK2D*. In summary, we have identified *C2dat2* as a novel I/R-induced lncRNA that acted similarly as *C2dat1* to mediate the upregulation of *CAMK2D* during I/R.

Next, murine neuronal cell survival was examined. Knockdown of *C2dat1* or *C2dat2* exacerbated OGD/R-induced neuronal cell death compared with the control (si-NT) in N2a (Fig. S2B) and murine primary neurons (Fig. 5d, e). Moreover, knockdown of both *C2dat1* and *C2dat2* resulted in greater cell death than knockdown each lncRNA alone, reaching approximately 60% cell death at 24 h post-OGD/R in N2a cells (Fig. S2B). Thus, *C2dat1* or *C2dat2* cooperated to promote neuronal survival by upregulating *CAMK2D* expression on post-ischemic neurons.

### CaMKIIδ Acted Through the NF-κB Signaling Pathway to Promote Neuronal Survival

**Fig. 6:**
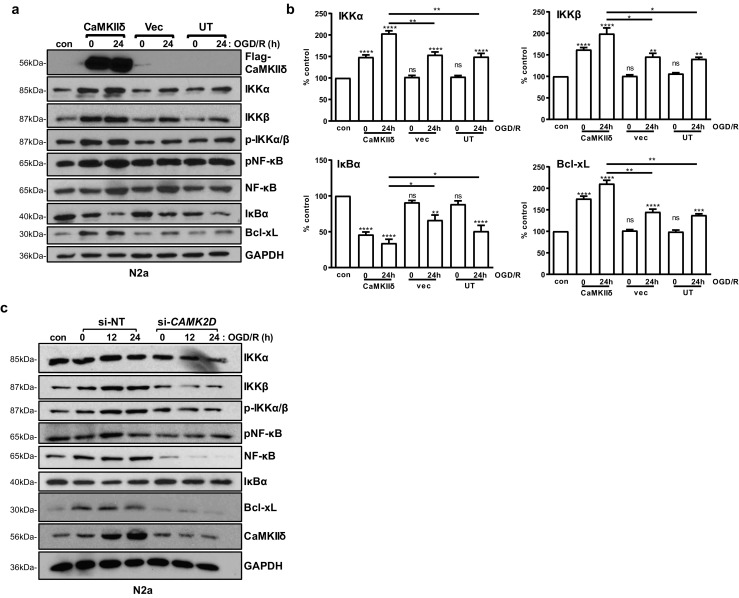
CaMKIIδ promoted neuronal survival through the NF-κB signaling pathway. **a** N2a cells were transfected with a Myc-DDK-CaMKIIδ plasmid. Twenty-four hours after transfection, the cells were subjected to OGD/R for 0 and 24 h. Cells cultured in normal medium under normoxic condition were used as controls (con). The cell lysates were analyzed by Western blotting as indicated. GAPDH was blotted as the loading control. Representative images from one of three independent experiments are shown. **b** Quantification of the levels of IKKα, IKKβ, IκBα, and Bcl-xL by densitometry analysis. Percent control (*y*-axis) represents the expression of the target genes to that of the controls under normoxic condition (equal to 100%). Data are the mean ± SEM from at least three independent experiments. ns, not significant; **P < 0.01; ***P < 0.001; ****P < 0.0001 vs. control by one-way ANOVA. con, control. **c** N2a cells were transfected with *CAMK2D* siRNA (si-*CAMK2D*) or si-NT. After 48 h, the cells were subjected to OGD/R for 0, 12, and 24 h. Cell lysates were analyzed by immunoblotting for members of the NF-κB signaling pathway. GAPDH was blotted as the loading control. Representative images from one of three independent experiments are shown

The signaling mechanisms of CaMKIIδ were examined by altering its expression in N2a cells. Although we had shown previously that *C2dat1*-mediated *CAMK2D* upregulation acts through the NF-κB signaling pathway [12], the function and signaling mechanism of *CAMK2D* per se have not been examined. Here, we showed that overexpression of CaMKIIδ promoted the activation of NF-κB signaling pathway. As shown in Figs. 6a, b and S3A, compared to the untransfected (UT) and empty vector transfected cells, overexpression of CaMKIIδ led to a marked elevation of IKKα, IKKβ, pS^176/180^-IKKα/β, p-NF-κB, NF-κB, and Bcl-xL (~ 2-fold), along with decreased IκB*α* at 24 h after OGD/R. These findings corroborated with the results obtained from knockdown of CaMKIIδ, which had the opposite effects on members of the NF-κB signaling pathways (Figs. 6c and S3B). Collectively, these data indicated that the NF-κB signaling pathway was a major pathway mediating the effects of CaMKIIδ during I/R.

### Knockdown of *C2dat1* and *C2dat2* Decreased CaMKIIδ Expression and Inhibited the NF-κB Signaling Activity. The NF-κB Signaling Pathway Also Acted Downstream of CaMKIIγ

**Fig. 7:**
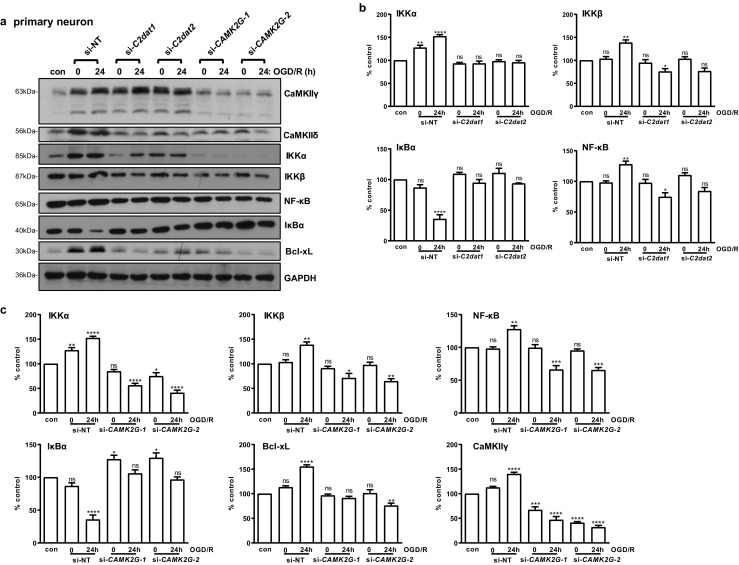
Knockdown of *C2dat1* and *C2dat2,* or *CAMK2G* inhibited the activation of the NF-κB signaling pathway. **a** Primary neurons were transfected with si-*C2dat1*, si-*C2dat2*, si-*CAMK2G*-*1*, si-*CAMK2G*-*2*, or si-NT, followed by OGD/R. Cells cultured in normal medium under normoxic condition were used as controls (con). Cell lysates were analyzed by immunoblotting for members of the NF-κB signaling pathway. GAPDH was blotted as the loading control. Representative images from one of three independent experiments are shown. **b** Quantification of the levels of IKKα, IKKβ, NF-κB, and Bcl-xL in cells with or without *C2dat1* or *C2dat2* knockdown by densitometry analysis. **c** Quantification of the IKKα, IKKβ, NF-κB, IκBα, Bcl-xL, and CaMKIIγ levels with or without *CAMK2G* knockdown. Percent control (*y*-axis) represents the expression of the target genes to that of the controls under normoxic condition (equal to 100%). Data in **b** and **c** are the mean ± SEM from three independent experiments. ns, not significant; *P < 0.05; **P < 0.01; ***P < 0.001; ****P < 0.0001 vs. control by one-way ANOVA. con, control

Our previous study confirmed that *C2dat1*-induced CaMKIIδ expression promoted neuronal survival through the activation of the NF-κB signaling pathway [12]. To determine if *C2dat2* acted through the same signaling mechanisms, both *C2dat1* and *C2dat2* were depleted in N2a cells and primary neurons followed by OGD/R. As shown in Figs. 7a and S4, OGD/R-induced *CAMK2D*/CaMKIIδ was blocked by knockdown of *C2dat1* and *C2dat2* in primary neurons (Fig. 7a, b) and N2a (Fig. S4). Meanwhile, IKKα, IKKβ, NF-κB, and Bcl-xL were also reduced, along with increased IκB*α*, indicating inactivation of the NF-κB signaling activity and downregulation of NF-κB-targeted genes (Bcl-xL) in rat primary neurons (Fig. 7a, b). Bcl-xS, an alternatively spliced Bcl-x variant, was not detected before or after OGD/R in primary neurons.

The effect of *CAMK2G* knockdown on the NF-κB signaling pathways was also examined in neurons. Our data showed that knockdown of *CAMK2G* similarly blocked the activation of the NF-κB signaling activity by downregulating IKKα, IKKβ, and NF-κB expression, blocking IκBα degradation, and reduced Bcl-xL expression in primary neurons. Meanwhile, knockdown of *C2dat1* and *C2dat2* blocked the upregulation of CaMKIIδ but did not affect the expression of CaMKIIγ (Fig. 7a, top lane), implying that the *C2dat* lncRNAs selectively targeted *CAMK2D*. Interestingly, knockdown of *CAMK2G* appeared not only to have depleted CaMKIIγ but also reduced the expression of CaMKIIδ (Fig. 7a). These results again verified that *C2dat1* and *C2dat2* specifically targeted *CAMK2D*, and both CaMKIIδ and CaMKIIγ signaled through the NF-κB signaling pathway in primary neurons.

### Loss of p65 NF-κB Facilitated Ischemia-Induced Neuronal Death and Decreased CaMKIIδ and CaMKIIγ Expression

**Fig. 8:**
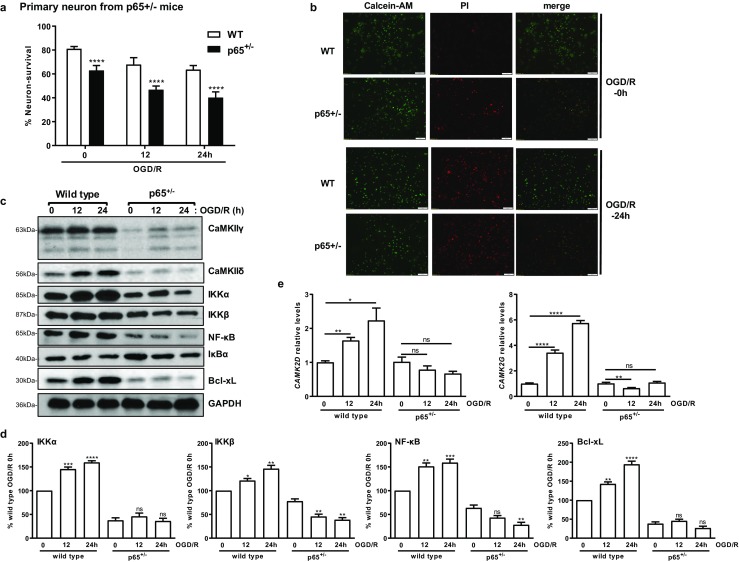
Knockout of NF-κB facilitated OGD/R-induced neuronal cell death, blocked the upregulation of CaMKIIδ, CaMKIIγ, and the activation of NF-κB signaling pathway. **a** Knockout of NF-κB increased OGD/R-induced cell death. Primary neurons from wild-type (WT) and p65 heterozygous mice (p65^+/−^) were obtained and subjected to OGD/R for 0, 12, and 24 h. Cell survival was assessed by calcein-AM/PI staining at 0, 12, and 24 h after OGD/R. The data are the mean ± SEM of three independent experiments. ****P < 0.0001 vs. WT by Student’s *t* test. **b** Representative images of calcein-AM/PI-stained primary neurons at 0 and 24 h post-OGD/R. red, PI; green, calcein-AM. **c** Primary neurons from WT and p65^+/−^ mice were subjected to OGD/R for 0, 12, and 24 h. Cell lysates were analyzed by immunoblotting for CaMKIIδ, CaMKIIγ, and members of the NF-κB signaling pathway. GAPDH was blotted as the loading control. Representative images from one of three independent experiments are shown. **d** Quantification of the IKKα, IKKβ, NF-κB, and Bcl-xL levels by densitometry. The mean ± SEM from three independent experiments were calculated and plotted. **e** OGD/R-induced *CAMK2D* and *CAMK2G* expression were abolished in p65^+/−^ mice. The data were measured by real-time RT-qPCR. The mean ± SEM of three independent experiments with triplicate determinations were calculated at each point. ns, not significant; *P < 0.05; **P < 0.01; ***P < 0.001; ****P < 0.0001 vs. 0 h control by unpaired *t* test

It has been demonstrated that cerebral ischemia activated the NF-κB signaling pathway; however, its functions in ischemic injury are variable, both pro- and anti-survival functions have been implicated. To further this investigation, primary neurons from heterozygous NF-κB knockout (p65^+/−^) mouse embryos [15] were prepared and subjected to OGD/R. As shown in Fig. 8a, b, loss of p65 NF-κB not only made the neurons more sensitive to ischemic treatment but also increased OGD/R-induced neuronal death as compared to the wild-type (WT) mice. Interestingly, the basal and OGD/R-induced *CAMK2D*/CaMKIIδ and *CAMK2G*/CaMKIIγ were significantly attenuated in p65^+/−^ NF-κB mice as compared to the WT mice (Fig. 8c–e). This was accompanied with the almost complete blockade of the NF-κB signaling activity, decreased IKKα and IKKβ, inhibition of IκBα degradation, and significantly downregulated Bcl-xL (Fig. 8c, d). These findings demonstrated a neuroprotective role of the NF-κB signaling pathway during I/R and a feedback regulation of CaMKIIδ/γ by NF-κB.

## Discussion

CaMKII as one of the most abundant protein kinase families in the CNS has long been implicated in cerebral ischemia, but its function in neuronal survival remains controversial. Meanwhile, the specific isoforms involved and their regulatory mechanisms are still largely unknown. This study aims at deciphering the function and signaling mechanisms of CaMKII kinases in ischemic damage in the brain. We reported for the first time selective induction of two CaMKII isoforms, namely CaMKIIδ and CaMKIIγ, by I/R. In particular, CaMKIIγ has not been associated with cerebral ischemia in the past. Our study also revealed novel lncRNA-mediated regulation of CaMKIIδ expression and identified *C2dat2* as a novel I/R-induced lncRNA, which cooperated with *C2dat1* to modulate the expression of *CAMK2D* in response to I/R. Our data further demonstrated that CaMKIIδ and CaMKIIγ activate the NF-κB signaling pathways, which acted to promote neuronal survival during I/R. The neuroprotective roles of CaMKIIδ and CaMKIIγ may benefit therapeutic intervention of I/R-induced neuronal injury.

It is a classical view that the predominant CaMKII isoforms in brain are CaMKIIα and CaMKIIβ, the former is the dominant form in forebrain, the latter in the cerebellum [16]. It is believed that CaMKIIδ and CaMKIIγ are ubiquitously distributed, but their levels in the brain are much lower than CaMKIIα and CaMKIIβ [3, 17–19]. Therefore, CaMKIIδ and CaMKIIγ have received less attention in the neuronal system. Here, for the first time, we demonstrated that I/R selectively induced both CaMKIIδ and CaMKIIγ in primary neurons. Using the pan-CaMKII antibody that recognizes all four isoforms of CaMKII, we found that CaMKIIδ, but not CaMKIIα or CaMKIIβ, was the predominant isoform expressed in N2a neuronal cells. In contrast, in murine primary neurons, CaMKIIγ was shown to be the most abundant isoform, followed by CaMKIIα and then CaMKIIδ/β. The low expression of CaMKIIδ/β in isolated neurons challenges the traditional view described above, although it is possible that this was attributed to the low reactivity of the pan-antibody towards CaMKIIδ/β. Nonetheless, the high expression of CaMKIIγ and its persistent upregulation by I/R was indeed a novel finding that implies a potentially important role of this isoform in neurons. Moreover, the fold of upregulation of *CAMK2G* was much greater than *CAMK2D* at the peak time (7- vs. 3~4-fold), although the difference was smaller at protein levels. Overall, these findings suggest that CaMKIIγ may be functionally important to neurons in the brain. We also further investigated the specific CaMKIIδ isoforms expressed in the neurons and N2a cells, and their response to OGD/R. Interestingly, we found that all four subtypes of CaMKIIδ were upregulated by OGD/R in N2a cells, while only CaMKIIδ_2_ was upregulated by OGD/R in neurons. The selective induction of CaMKIIδ_2_ is intriguing, which could be due to (1) different subtypes of CaMKIIδ was expressed in N2a and primary neurons with CaMKIIδ_2_ being the predominant form in neurons (others are minimally expressed); (2) the mechanism that mediated the upregulation of CaMKIIδ_2_ may be different in the neurons as compared to N2a, although knockdown of *C2dat1* or *2* did not differentially impact the subtypes induced by OGD/R in neurons vs. N2a cells (data not shown). This (difference in CaMKIIδ subtypes induced by OGD/R in neurons vs. N2a) may also explain the different levels of CaMKIIδ detected by pan-CaMKII antibody in N2a vs. primary neurons, since the antibody may preferentially recognize one subtype of CaMKIIδ but not the others. To test this, we re-examined the levels of CaMKIIδ using a CaMKIIδ-specific antibody (presumably recognizing most subtypes of CaMKIIδ). This antibody was able to detect CaMKIIδ with greater sensitivity and demonstrated high expression of CaMKIIδ in neurons as well as N2a (Fig. S1). In contrast, *CAMK2A*/CaMKIIα and *CAMK2B*/CaMKIIβ were not induced by OGD/R, which is in line with the finding that activated CaMKIIα did not play any significant role in regulating ischemic cell death [20]. Taken together, our study demonstrated high expression and selective induction of CaMKIIδ and CaMKIIγ by I/R in neuronal cells. These findings have identified CaMKIIδ and CaMKIIγ as new players in ischemic injury in the brain.

Our previous study reported the discovery of *C2dat1*, which was induced by I/R in murine MCAO models and neuronal cells [12]. In this study, we described the identification of another novel *CAMK2D*-associated lncRNA - *C2dat2*, which had similar function as *C2dat1* despite targeting a different region of the *CAMK2D* gene. Knockdown of *C2dat1* and/or *C2dat2* increased neuronal cell death and selectively abolished OGD/R-induced *CAMK2D*/CaMKIIδ upregulation, but did not affect the expression of *CAMK2G* gene and CaMKIIγ protein, implying this regulation is likely isotype specific as it exclusively targets *CAMK2D*, and there are no overlapping sequences between *CAMK2D* and those of *CAMK2A*, *2B*, and *2G*.

Ischemic insult to the brain not only causes progressive neuronal cell death but also induces adaptive responses which act to reduce damage and promote neuronal survival. Mechanisms underlying these prosurvival adaptive responses are not fully understood. Our study has identified the NF-κB pathway as one of the major signaling pathways activated downstream of CaMKIIδ and CaMKIIγ. We showed that increased NF-κB signaling activity conferred neuroprotection in N2a and murine primary neurons. NF-κB is a master regulator of inflammation and immune responses [21]. It is potently activated in brain tissues from stroke patients and ischemic insulted animals [22]. The activation of NF-κB induces a variety of genes that could either promote or inhibit cell survival [23]. Although many studies have identified the NF-κB pathway as a pro-inflammatory signaling pathway to promote neuronal cell death in response to injury, neuroprotective role of NF-κB has also been reported in ischemic injury [24]. The pro-survival effect of NF-κB in neurons is mediated through the upregulation of several antiapoptotic proteins, including superoxide dismutase [25], Bcl-2, and Bcl-xL [26]. Studies have shown that the spatial and temporal control of NF-κB activation may determine whether it promotes neuronal death or survival [22]; however, the precise mechanisms underlying its effect in ischemic injury remains to be fully defined [24, 27–29]. Using neurons obtained from p65^+/−^ knockout mice, we showed the knockdown of NF-κB p65 moderately exacerbated OGD/R-induced neuronal cell death. The loss of p65 also resulted in downregulation of CaMKIIδ and CaMKIIγ, implying NF-κB p65 may feedback regulating the expression of CaMKIIδ and CaMKIIγ. This could be a mechanism that maximizes the neuroprotective activity of this signaling pathway. Further analysis is needed to reveal the underlying mechanisms.

In summary, our study has demonstrated I/R-induced selective upregulation of CaMKIIδ and CaMKIIγ and revealed important mechanisms that mediated the upregulation of CaMKIIδ. Our functional analysis demonstrated the neuroprotective role of both CaMKIIδ and CaMKIIγ in neuronal cells through activation of the NF-κB signaling pathway. This study sheds lights upon mechanisms of neuroprotection and recovery following cerebral ischemia, and provided novel molecular targets and signaling pathways that may be exploited for treatment of ischemic injuries in the brain.

## Materials and Methods

### Animals and Primary Cortical Neuron Preparation

Wild type C57BL/6J mice at 8–10 weeks old (25–30 g body weight, male, female) were used for middle cerebral artery occlusion (MCAO). Animals were housed in a temperature- and humidity-controlled animal facility with a 12-h light-dark cycle. Food and water were available ad libitum. The NF-κB p65 knockout mouse colony was described previously [30]. Genotyping of p65 knockout mice was described in SI Materials and Methods. All animal experiments were approved by the University of Pittsburgh Institutional Animal Care and Use Committee and performed in accordance with the National Institutes of Health Guide for the Care and Use of Laboratory Animals. For preparation of rat primary cortical neurons, embryonic day 18 (E18) wild-type Sprague-Dawley rat pups, E15–17 wild-type C57BL/6J mouse pups, or E15–17 NF-κB p65 knockout mouse pups were used for primary cortical neuron preparation and culture as described previously [31]. Details of primary neuron preparation are given in SI Materials and Methods.

### MCAO Surgery

Transient focal cerebral ischemia was induced in mice by intraluminal occlusion of the left middle cerebral artery (MCAO) for 50 min as described previously [31]. Details of MCAO surgery and tissue collection and processing are provided in SI Materials and Methods.

### Cell Lines, In Vitro Ischemia by Oxygen-Glucose Deprivation/Reoxygenation (OGD/R), and Cell Survival Assays

N2a cells (ATCC) and primary cortical neurons were subjected to in vitro ischemia by oxygen and glucose deprivation for 3 h (N2a) or 1 h (primary neurons), followed by incubation in glucose-containing medium under normoxic conditions for various times [32]. N2a cells survival was assayed using Cell Counting Kit-8 (Dojindo Laboratories, Kumamoto, Japan) [12]. Primary neuronal cell viability was assessed by calcein-AM/propidium iodide (PI) staining assay as previously described [31, 33]. Details of cell culture, OGD/R, and cell survival assays are provided in SI Materials and Methods.

### Plasmid and siRNA Transfection, RNA Extraction, Real-Time RT-qPCR, Western Blotting Analysis

Myc-DDK-CaMKIIδ plasmid (OriGene) plasmid and *CAMK2D*- or *CAMK2G*-targeted siRNAs were transfected using standard procedures according the manufacturers’ instructions. Total RNAs from tissues or cells were extracted using TRIzol LS Reagent or RNeasy mini kit (Qiagen). Sequences of real-time polymerase chain reaction (PCR) primers are provided in Table S1. Details of transfection procedures, RNA extraction, real-time RT-qPCR, and Western blotting analysis are given in SI Materials and Methods.

### Statistical Analysis

All measurements were performed by investigators who were blinded to the experimental conditions. Data was analyzed by using the Student’s *t* test for comparison between two groups (two-tailed) and one-way or two-way ANOVA for multiple groups followed by Bonferroni’s multiple comparisons test. All statistical analysis was done using GraphPad Prism IV software (GraphPad Software, La Jolla, CA, USA). All values are shown as the mean ± SEM of at least three independent experiments. A *p* value < 0.05 was considered statistically significant (*p < 0.05; **p < 0.01; ***p < 0.001; ****p < 0.0001; ns, not significant).

## Electronic supplementary material

ESM 1 (PDF 1205 kb)

## Abbreviations

PCR: polymerase chain reaction
LncRNA: long non-coding RNA
I/R: ischemia/reperfusion
C2dat1: *CAMK2D*-associated transcript 1
C2dat2: *CAMK2D*-associated transcript 2
OGD/R: oxygen-glucose deprivation/reoxygenation
CNS: central nervous system
CaMKII: calcium/calmodulin-dependent kinase II
NF-κB: nuclear factor-κB
IKK: IκB kinase complex
IκB: inhibitory κB
MCAO: middle cerebral artery occlusion
PI: propidium iodide
N2a: mouse Neuro 2A
si-NT: non-targeting siRNA
