# Supplementary material for: Ischemic Injury-Induced CaMKIIδ and CaMKIIγ Confer Neuroprotection Through the NF-κB Signaling Pathway
Source: Mol Neurobiol. 2018 Jul 11;56(3):2123–36. doi: 10.1007/s12035-018-1198-2 (PMC6394630; doi:10.1007/s12035-018-1198-2)
Supplement: Supplementary file 1 — (PDF 1205 kb) [file 12035_2018_1198_MOESM1_ESM.pdf]

Supplemental Figures and Tables

SI Figure Legends

Figure S1

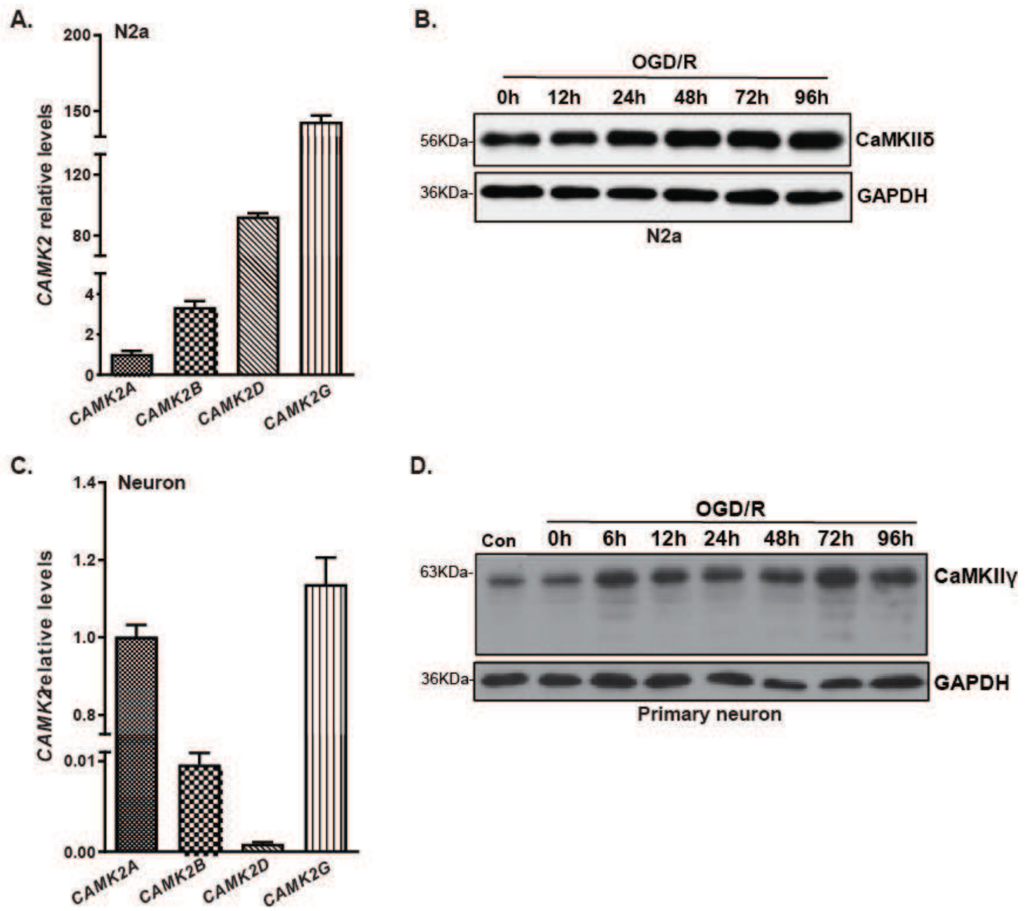

Figure S1. Expression of *CAMK2* isoforms and OGD/R induced *CAMK2D*/CaMKIIδ upregulation in N2a and murine neurons. N2a cells (A) and rat primary neurons (C) were collected and analyzed by real-time RT-qPCR for *CAMK2A*, *CAMK2B*, *CAMK2D*, and *CAMK2G* transcripts. The data are the mean ± S.E.M. of three independent experiments with triplicate determinations at each point. (B) Western blot analysis with anti-CaMKIIδ antibody showed that CaMKIIδ expression was significantly elevated in response to OGD/R in N2a cells in a time-dependent manner. (D) Rat primary neurons were subjected to OGD/R, followed by immunoblotting using the pan-CaMKII antibody (a short exposure showing the upregulation of CaMKIIγ). Representative images from one of at least three independent experiments are shown.

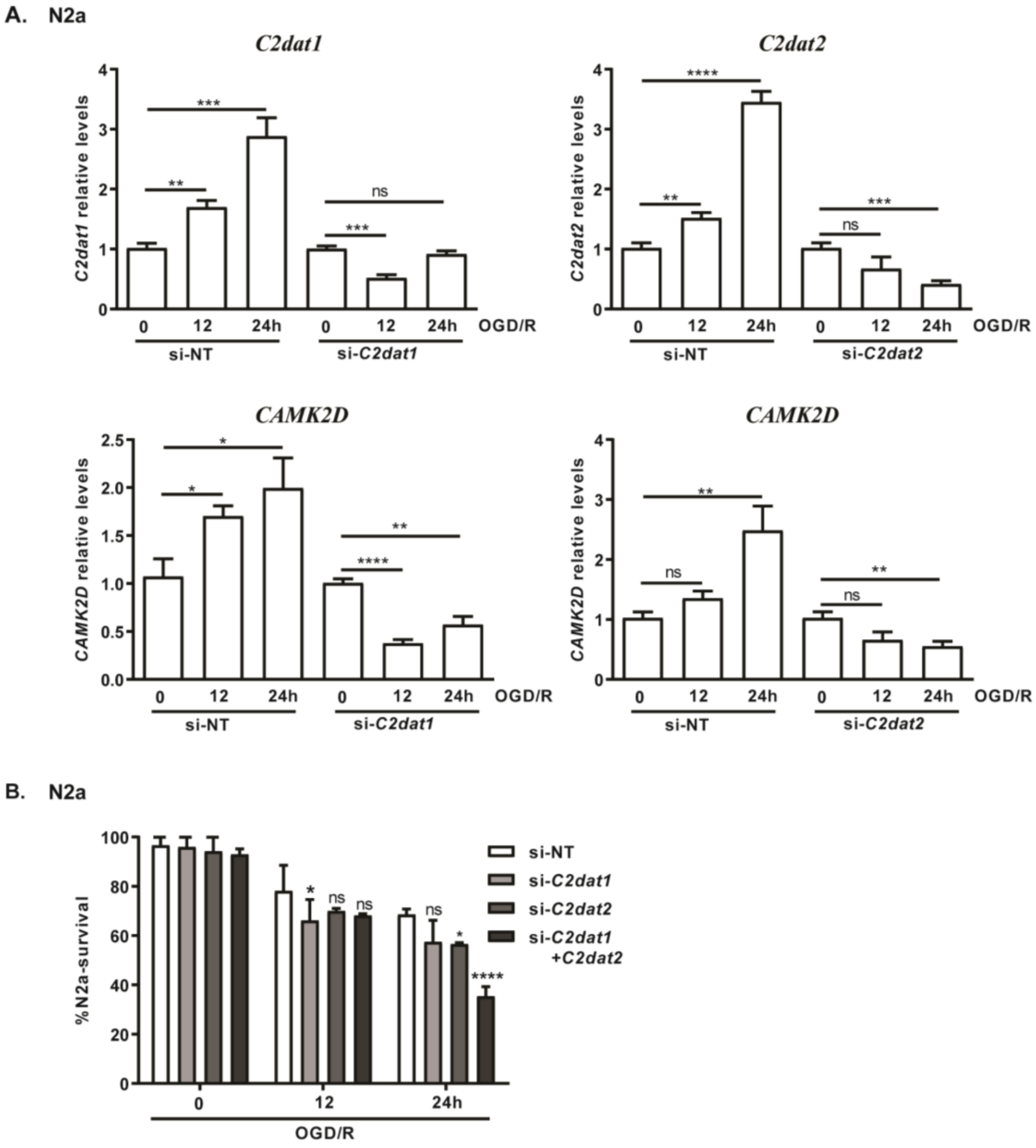

Figure S2. Knockdown of *C2dat1* or *C2dat2* inhibited OGD/R-induced upregulation of *C2dat1* or *C2dat2*, and abolished OGD/R-induced *CAMK2D* expression in N2a cells. (A) LncRNA *C2dat1*- or *C2dat2*-targeted siRNA (si-*C2dat1*, si-*C2dat2*) and the non-targeting siRNA (si-NT) were transfected into N2a cells. 48 h after transfection, the cells were subjected to OGD/R for 0, 12, 24 h. The cells were collected and analyzed by real-time RT-qPCR for *C2dat1, C2dat2*, and *CAMK2D* transcripts. The data are the mean ± S.E.M. of three independent experiments with triplicate determinations at each point. ns, not significant; *$P<0.05$; **$P<0.01$; ***$P<0.001$; ****$P<0.0001$ vs. 0 h control by unpaired *t*-test. (B) Knockdown *C2dat1* and/or *C2dat2* exacerbated neuronal cell death after OGD/R. N2a cells were transfected with si-*C2dat1*, si-*C2dat2*, or si-NT, followed by OGD/R for 0, 12, 24 h. Cell survival was measured by CCK-8 assay. The data are the mean ± S.E.M. of at least three independent experiments with triplicate determinations at each point. ns, not significant; *P<0.05; ****P<0.0001 vs. si-NT by unpaired t-test.

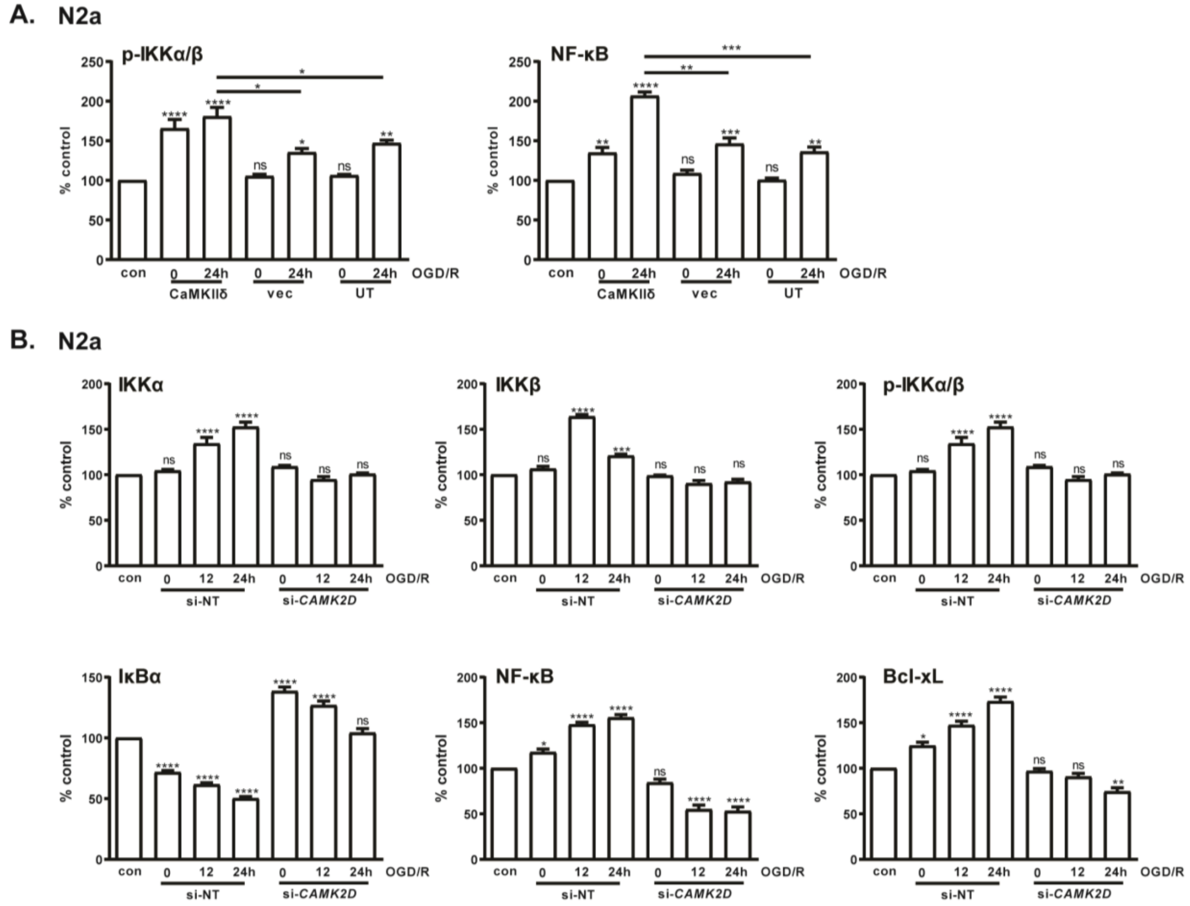

Figure S3. Overexpression of CaMKIIδ promoted, while knockdown of CaMKIIδ inhibited, the activation of the NF-κB signaling pathway by OGD/R in N2a cells. (A) Quantification of p-IKKα/β and NF-κB levels in "Fig. 6A" by densitometry analysis. N2a cells were transfected with a Myc-DDK-CaMKIIδ plasmid, and then subjected to OGD/R for 0 and 24 h, followed by Western blotting. (B) Quantification of IKKα, IKKβ, p-IKKα/β, NF-κB, IκBα and Bcl-xL expression in "Fig. 6C" by densitometry analysis. N2a cells were transfected with *CAMK2D* siRNA (si-*CAMK2D*). The Western blots from at least three independent experiments were evaluated by densitometry analysis and the mean ± S.E.M. from these data were calculated and plotted. ns, not significant; **P*<0.05; ***P*<0.01; ****P*<0.001; *****P*<0.0001 vs. control by One-Way ANOVA. con, control.

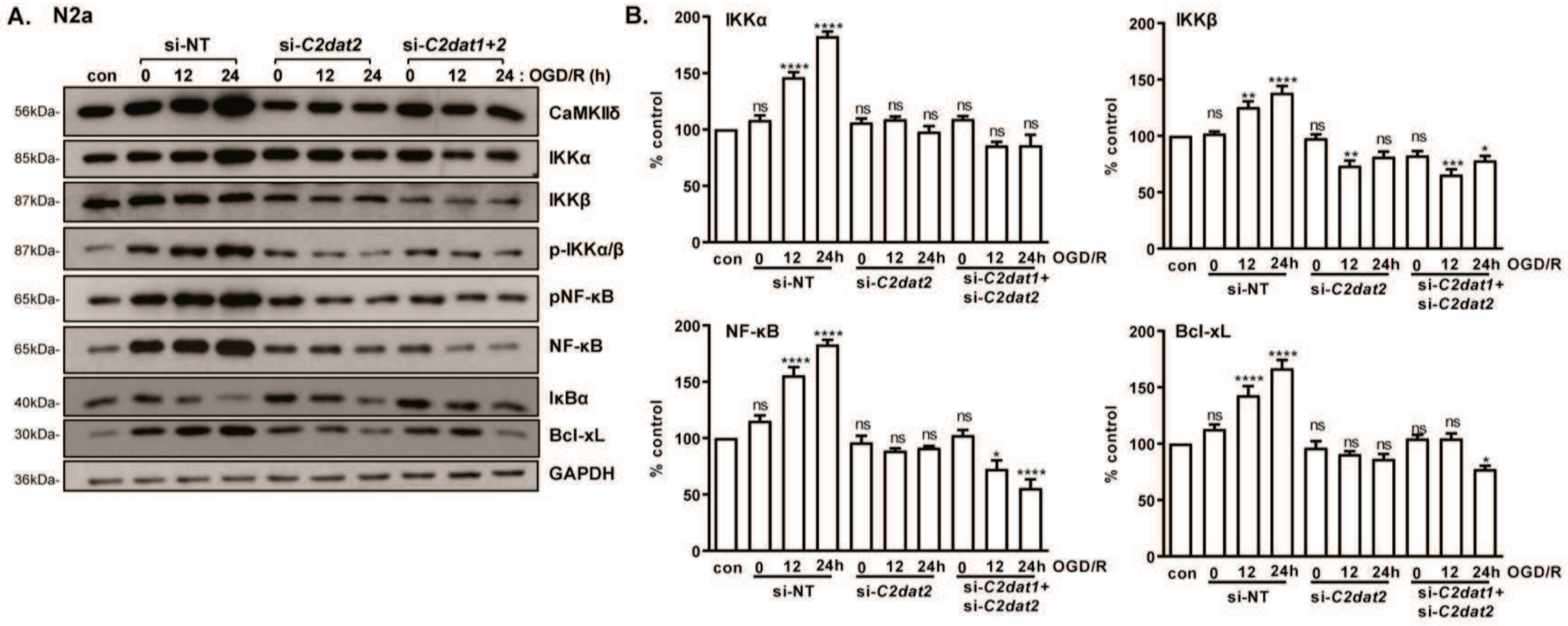

Figure S4. Knockdown of *C2dat2* or *C2dat1/2* inhibited the activation of the NF-κB signaling pathway in N2a cells. (A) Knockdown of *C2dat2* (si-*C2dat2*) or both *C2dat1* and *C2dat2* (si-*C2dat1+2*) blocked the upregulation of CaMKIIδ and the activation of the NF-κB signaling pathway induced by OGD/R in N2a cells. N2a cells were transfected with si-*C2dat2,* si-*C2dat1* +si-*C2dat2*, or si-NT. Two days after transfection, the cells were subjected to OGD/R. Representative images from one of at least three independent experiments were shown. (B) Quantification of the levels of IKKα, IKKβ, NF-κB and Bcl-xL by densitometry analysis. The mean ± S.E.M. from three independent experiments were calculated and plotted. ns, not significant; *P<0.05; **P<0.01; ***P<0.001; ****P<0.0001 vs. control by One-Way ANOVA. con, control.

Table S1. List of real time PCR primers used in the study.

Mouse lncRNA or mRNA	Forward primer (5′–3′)	Reverse primer (5′–3′)
AK153573 (C2dat1)	ATGTAAGGGCTTGGTCCCAC	TGTAGCCTCCGTGTGAAAGG
AK020214 (C2dat2)	CGTCTCATGAAGCACCCCAA	CAACCATGATCAAGAGACTAGGA
CAMK2D	TATCGGAGGAGGGCTTCCAT	ACTGGCATCAGCTTCACTGT
CAMK2A	CCATCCTCACCACTATGCTG	ATCGATGAAAGTCCAGGCCC
CAMK2B1	TGCAAGGAGGAAGCTCAAGG	ATCCTTTGGGGCTGGTGATG
CAMK2B2	CCACCGTGGCCTCTATGATG	GGGCTTGACTCCATCTGCTT
CAMK2G1	GGGTCTGTCAACGGTCTACG	GACACCGCCATCTGACTTCT
CAMK2G2	TTACGCAAATTCAACGCCCG	ACCGCCATCTGACTTCTTGT
CAMK2D-3	CGAGAAATTTTTCAGCAGCC	GCTCTCAGTTGACTCCATCATC
CAMK2D-2	CGAGAAATTTTTCAGCAGCC	CTCAGTTGACTCCTTTACCCC
GAPDH	GGACCTCATGGCCTACATGG	TAGGGCCTCTCTTGCTCAGT
CAMK2D-1/4	CAACAAAGCCAACGTGGTAACC	GTGCTTTCACGTCTTCATCCTC
CAMK2D-2/3	AAGACCATGCAGTCAGAAGAGACG	GTTTTGCCACAAAGAGGTGC
Rat lncRNA or mRNA	Forward primer (5′–3′)	Reverse primer (5′–3′)
C2dat1	ATATAAGGGCTTGGTCCCAC	TGTAGCCTCTATGTTAAAGG
C2dat2	CGTCTCATGAAGCACCCCAA	GCGTGATGAGAGACTAGAACCT
CAMK2D	GCTCGACGGAAATTGAAGGG	TCTGAACACTCGAACTGGACT
CAMK2A	CCTGTATATCTTGCTGGTTGGG	TTGATCAGATCCTTGGCTTCC
CAMK2B	TCAAGCCCCAGACAAACAG	GAGATCCTGGGGGCTTTGG
CAMK2G	AAACCTGTGGATATCTGGGC	CTGGTGATGGGAAATCGTAGG
GAPDH	CAAGGTACTCCATGACAACTTTG	GGGCCATCCACAGTCTTCTG

Table S2. List of antibodies used in the study.

antibody	Catalog number	dilution
Anti-CaMKIIδ antibody	Badrilla Inc **A010-55AP**	1:5000
CaMKII (pan) (D11A10) Rabbit antibody	Cell Signaling #4436	1:1000
GAPDH antibody	Cell Signaling #97166	1:5000
Anti-flag antibody	Cell Signaling #14793	1:1000
p44/42 MAPK (Erk1/2) antibody	Cell Signaling #4695	1:3000
Phospho-p44/42 MAPK (Erk1/2) antibody	Cell Signaling #4370	1:1000
Akt antibody	Cell Signaling #9272	1:3000
Phospho-Akt (Ser473) antibody	Cell Signaling #4060	1:1000
PI3K (p85) antibody	Cell Signaling #4292	1:1000
NFκB Pathway Sampler Kit	Cell Signaling #9936	1:1000
Bcl-xL antibody	Cell Signali#2762	1:1000

SI Materials and Methods

Reagents and antibodies

The iScript cDNA synthesis kit, EVA Green SMX 500R, goat anti-rabbit and goat anti-mouse HRP-conjugated secondary antibodies were obtained from Bio-Rad Laboratories (Richmond, CA). Cell culture reagents and media were from American Type Culture Collection (ATCC, Rockville, MD) and Thermo Fisher Scientific (Pittsburgh, PA). Anti-CaMKIIδ antibody was purchased from Badrilla Ltd (Leeds, UK). GAPDH antibody, CaMKII (pan) antibody, Flag antibody, p44/42 MAPK (Erk1/2) antibody, Phospho-p44/42 MAPK (p-Erk1/2) antibody, Akt antibody, Phospho-Akt (Ser473) antibody, PI3K antibody, the NFκB Pathway Sampler Kit, and Bcl-xL antibody were purchased from Cell Signaling Technology (Danvers, MA). Please see Table S2 for a list of antibodies used in the study.

Genotyping of p65 Knockout Mice

Mouse genotyping was conducted by polymerase chain reaction (PCR) using REDExtract-N-Amp™ Tissue PCR Kit according to the manufacturers' instructions (Sigma, St Louis, Missouri) (15). Mouse livers were cut into pieces (2–3 mm) and the mixtures of extraction solution and tissue preparation solution were added. The samples were incubated at room temperature for 30 min and then boiled for 3 min. The neutralization solution buffer was then added. The PCR reaction was set up by mixing 10 µl REDExtract-N-Amp Tissue PCR Red Mix, 6 µl of the primers, and 4 µl samples. The PCR primers for p65 were: CCTATAGAGGAGCAGCGCGGG (sense), AATCGGATGTGAGAGGACAGG (antisense for WT), AAATGTGTCAGTTTCATAGCCTGAAGAACG (antisense for p651/2). The thermal cycling conditions were as follows: an initial denaturation step at 94 °C for 5 min; 35 cycles including denaturation at 94 °C for 45 s; annealing at 54 °C for 45 s; extension at 72 °C for 90 s; and then a final extension at 72 °C for 5 min. The amplified PCR product was analyzed by standard agarose gel electrophoresis.

MCAO surgery

Transient focal cerebral ischemia was induced in mice by intraluminal occlusion of the left middle cerebral artery (MCAO) for 50 min as described previously (31). Achievement of ischemia was confirmed by monitoring regional cerebral blood flow with laser speckle contrast imager (Pericam). For reperfusion, the suture was withdrawn after the MCAO. Sham-operated mice underwent the similar operations to expose the carotid arteries without the suture insertion. After recovery, animals were returned to their cages with free access to food and water. Mice were sacrificed at 24h, 48h, 72h and 96h after reperfusion. Mouse brains were removed quickly for biochemical analysis. Part of the brain tissue was snap frozen in isopentane for cryostat sectioning. Serial coronal sections (1 mm apart) were stained with 2, 3, 5-triphenyltetrazolium chloride monohydrate (TTC; Sigma-Aldrich, St. Louis, MO) for assessing size of infarction. Tissues from the ischemic core and surrounding penumbra were isolated and snap frozen in liquid N2 for western blotting analysis.

Primary cortical neuron preparation and culture

The primary neuron culture was prepared as described previously (31). The pregnant rats and mice were anesthetized with 5% isoflurane and euthanized. Fetuses were removed and the cortices dissected from individual pups in ice-cold Hank's Balanced Salt Solution. The tissues

were treated with 200 Units papain solution at 37°C for 4 min followed by 3 washes with trypsin inhibitor at 37°C for 4 min each. Tissues were mechanically dissociated through pipetting up and down several times until no obvious particles were seen. The cells were then plated at a density of 60 × 10^3 cells/cm^2 in Poly-D-Lysine-coated culture dishes with neurobasal medium containing B-27 supplements, L-Glutamine and penicillin/streptomycin (100 U/mL / 0.1 mg/mL). Neuronal Cultures were incubated at 37°C in an incubator with 5% CO_2 and neurons grown for 8-10 days were used in the study.

Cell lines and cell culture
Mouse Neuro 2A (N2a) cells were purchased from American Type Culture Collection (ATCC, Manassas, VA) and cultured in Minimum Essential Medium Eagle (MEM) with Eagle's salt and L-glutamine supplemented with 10% FBS (Invitrogen, Carlsbad, CA) at 37 °C with 5% CO_2.

In vitro ischemia by oxygen-glucose deprivation/reoxygenation (OGD/R)
To mimic ischemic-like conditions *in vitro*, the growth medium was replaced with deoxygenated glucose-free Hanks Balanced Salt Solution. Cells were placed into a temperature-controlled (37±1°C) anaerobic chamber (Forma Scientific, Marietta, OH) that contains a gas mixture composed of 1% O_2, 5% CO_2 and 94% N_2 for 3 h (N2a) or 1 h (primary neuronal cells). After OGD exposure, the medium was replaced with the original glucose-containing medium and cultured under normoxic condition for different times at 37 °C with 5% CO_2. Control cell cultures were not deprived of oxygen and glucose and always placed in normoxic glucose-containing medium, or harvest immediately after OGD treatment (t=0 min). OGD conditions were optimized to ensure cell viability before (con) and immediately after OGD (t=0 min) does not differ more than 5-10%.

Cell Survival Assays
N2a cells survival was assayed by Cell Counting Kit-8 (Dojindo Laboratories, Kumamoto, Japan), according to the manufacturers' instructions. Briefly, N2a cells were plated at a density of 1 × 10^5 cells per well in 24-well plates. After siRNA transfection and/or drug treatment, CCK-8 solution containing a highly water-soluble tetrazolium salt WST-8 [2-(2-methoxy-4-nitrophenyl)-3-(4-nitrophenyl)-5-(2,4-disulfophenyl)-2H-tetrazolium, monosodium salt] was added to cells in each well, followed by incubation for 2 h. Cell proliferation/viability was determined by measuring the OD at 450 nm. Percent over control was calculated as a measure of cell viability.
Primary neuronal cell viability was assessed by propidium iodide (PI) uptake and retention of calcein-AM based on the methods as previously described (31, 33). Cultured neurons were washed with 1xPBS and incubated with 20 nM calcein-AM viability dye (Invitrogen, Carlsbad, CA) and 1 µg/ml PI in PBS at 37°C for 30 min. Images were captured under an Olympus 1X71 inverted system microscope with 10X objective lens, and the Calcein-AM or PI stained cells were analyzed by Image J software (NIH, Bethesda, MD). Cells were counted in at least 10 random fields in each image. Cell viability was expressed as the ratio of calcein-AM-positive cells to the sum of calcein-AM-positive and PI-positive cells.

Plasmid and siRNA transfection
Myc-DDK-CaMKIIδ plasmid was obtained from OriGene Technologies (Rockville, MD) was transfect with Lipofectamine 3000 reagent (Invitrogen, Carlsbad, CA). For primary neuron cells,

the P3 Primary Cell 4D-Nucleofector reagent was used for siRNAs or plasmid transfection according to the manufacturers' instructions (Lonza, Walkersville, MD). The DsiRNAs of *CAMK2D* and *CAMK2G*, *C2dat1*- and *C2dat2*-targeted siRNAs, and a control non-targeting siRNA (negative control) were from Integrated DNA Technologies (IDT, Coralville, IA). The siRNAs were transfected into N2a cells using DharmaFECT transfection reagent (Lafayette, CO) or Cell Line Nucleofector V reagent (Lonza Walkersville, MD) according to the manufacturers' instructions. 24h or 48 h after transfection, N2a cells and primary neurons were subjected to OGD treatment, followed by reoxygenation under normoxic culture conditions for various times (0, 12 and 24 h).

Preparation of samples and RNA extraction
Total RNAs from N2a or primary neurons were extracted using TRIzol LS Reagent or RNeasy mini kit (QIAGEN, Germantown, MD). OD260 and OD280 of total RNA were measured by Synergy H1 Hybrid Multi-Mode Microplate Reader (BioTek, Winooski, VT). RNA purity was evaluated based on OD260/280 ratio and RNA integrity was assessed using standard denaturing agarose gel electrophoresis. The total amount of RNA obtained did not differ significantly between the samples.

Primers
A list of real-time PCR primers used in the study and their sequences are provided in Table S1.

Real-time RT-qPCR
cDNA synthesis from 1 μg total RNAs from N2a cells or 0.3 μg of total RNAs from primary neurons were carried out using the iScript cDNA synthesis kit (Bio-Rad, Richmond, CA). The levels of *C2dat1*, *C2dat2*, *CAMK2A, CAMK2B, CAMK2G* and *CAMK2D* transcripts was analyzed by real-time PCR using the SsoFast EvaGreen Supermix on CFX96 Real-Time PCR Detection System (Bio-Rad, Richmond, CA). Data were normalized using GAPDH as an internal control.

Western blotting analysis
Cells were harvested and lysed in lysis buffer (50 mM Tris-HCl, pH 7.4, 150 mM NaCl, 1.5 mM $MgCl_2$, 10% glycerol, 1% Triton X-100, 5 mM EGTA, 20 μM leupeptin, 1 mM AEBSF, 1 mM NaVO3, 10 mM NaF and 1 × protein inhibitor cocktail). The brain tissue was homogenized with Dounce hand homogenizers and lysed in homogenization buffer (40 mM Tris, pH 7.4, 2mM EDTA, 640mM Sucrose, 40mM Na pyrophosphate, 20mM NaF, 40mM β-glycerophosphate, 0.4 mM $NaVO_3$, and 1x protein inhibitor cocktail). Western blot analysis was carried out according to standard procedures. Briefly, 15 μg of proteins were separated on SDS-PAGE and transferred onto nitrocellulose membranes. After pre-blocking, the membranes were incubated with the appropriate primary antibody at 1:1000 dilutions at 4 °C overnight. After washing, the membranes were incubated with secondary antibody at room temperature for 1 h. Proteins were detected with the enhanced chemiluminescence (ECL) kit (Thermo Fisher). The images were captured on X-ray films and band intensity was quantified by Image J software (NIH, Bethesda, MD).
